# Effects of shortened host life span on the evolution of parasite life history and virulence in a microbial host-parasite system

**DOI:** 10.1186/1471-2148-9-65

**Published:** 2009-03-25

**Authors:** Thibault Nidelet, Jacob C Koella, Oliver Kaltz

**Affiliations:** 1 UPMC University Paris 06, Laboratoire de Parasitologie Evolutive – UMR 7103, 7 quai St-Bernard, 75252 Paris, France; 2 UMR de Génétique Végétale, University Paris Sud/INRA/CNRS/INAPG, Ferme du Moulon, 91190 Gif-sur-Yvette, France; 3 Division of Biology, Imperial College London, Silwood Park Campus, Ascot SL5 7PY, UK; 4 Institut des Sciences de l'Evolution, UMR 5554, Université de Montpellier 2, CC 065, Place Eugène Bataillon, 34095 Montpellier, France

## Abstract

**Background:**

Ecological factors play an important role in the evolution of parasite exploitation strategies. A common prediction is that, as shorter host life span reduces future opportunities of transmission, parasites compensate with an evolutionary shift towards earlier transmission. They may grow more rapidly within the host, have a shorter latency time and, consequently, be more virulent. Thus, increased extrinsic (i.e., not caused by the parasite) host mortality leads to the evolution of more virulent parasites. To test these predictions, we performed a serial transfer experiment, using the protozoan *Paramecium caudatum *and its bacterial parasite *Holospora undulata*. We simulated variation in host life span by killing hosts after 11 (*early *killing) or 14 (*late *killing) days post inoculation; after killing, parasite transmission stages were collected and used for a new infection cycle.

**Results:**

After 13 cycles (≈ 300 generations), parasites from the *early-killing *treatment were less infectious, but had shorter latency time and higher virulence than those from the *late-killing *treatment. Overall, shorter latency time was associated with higher parasite loads and thus presumably with more rapid within-host replication.

**Conclusion:**

The analysis of the means of the two treatments is thus consistent with theory, and suggests that evolution is constrained by trade-offs between virulence, transmission and within-host growth. In contrast, we found little evidence for such trade-offs across parasite selection lines within treatments; thus, to some extent, these traits may evolve independently. This study illustrates how environmental variation (experienced by the host) can lead to the evolution of distinct parasite strategies.

## Background

Understanding the factors that shape the evolution of parasite life history and virulence is a major issue in evolutionary biology [1-4], with important implications in applied and medical contexts [5,6]. According to standard theory, a parasite needs to exploit the host to increase its rate of transmission. If exploitation harms the host, the parasite must therefore trade current for future transmission and evolution should lead to the balance between virulence and the rate of transmission that maximizes the parasite's life-time reproductive success. Where this balance lies may depend on genetic [7], epidemiological [8-11] or environmental factors [12,13].

A general prediction concerning environmental factors is that increased extrinsic host mortality (i.e. the mortality that is not due to the parasite) selects for higher virulence [[11,14,16,16], but see [17]]. Because shorter host life span reduces future opportunities of transmission, the constraint to keep the host alive is relaxed. Rather, to compensate for the loss of future transmission, parasites should evolve to grow more rapidly within the host, start transmission earlier and, consequently, be more virulent. This prediction can be restated in terms of classical life-history theory: if future reproduction is compromised, we expect selection for earlier age at maturity and increased investment in early reproduction [18]. In this sense, the change in virulence reflects a shift in optimal latency time. A theoretical framework based on this life-history perspective has been developed for the evolution of lysis time in bacteriophages [19]. Such a framework is relevant for medicine, as, from the parasite's point of view, shorter host life span is similar to shortening the infection by the application of a drug treatment. It is therefore important to understand how parasite life-history, such as latency time or fecundity, responds to selection imposed by such a treatment [20,21].

Few experimental studies have investigated the effects of shortened host life span [22-25]. In a serial passage experiment, Cooper et al [22] directly manipulated host life span and timing of transmission of an insect virus by killing infected hosts at two time points after infection. Consistent with theory, early killing selected for higher virulence, possibly caused by more rapid within-host growth. In a similar type of experiment, infecting hamster cells *in vitro *with vesicular stomatitis virus, Elena [23] found that earlier transmission schedules selected for higher viral population growth rate and increased longevity of viral propagules in the medium, conferring a selective advantage during the early phase of an epidemic. A more complex picture was found in a serial transfer experiment on nematode parasite of rats [25]: with increasing numbers of nematode females inside the host, females from the early killing treatment became less fecund than those from the late killing treatment. Although it was not measured in this study, lower fecundity may also lead to a reduction in virulence. Finally, a clear-cut counterexample to theory was obtained in an experiment on the water flea, *Daphina magna*, and a microsporidian gut parasite [24]. In the treatment where host death rate was increased, parasites evolved to produce fewer transmission stages and were less virulent than parasites from the control treatment, possibly because the high mortality treatment led to a concomitant increase in the frequency of multiple infections [24]. Thus, these experiments show that parasites indeed respond to selection imposed by a shorter host life-span, but not always by increasing their virulence, as predicted by standard theory. In particular, it remains largely unclear how primary targets of selection, parasite latency time and age at maturity, evolved in these experiments [but see [22]].

**Figure 1:**
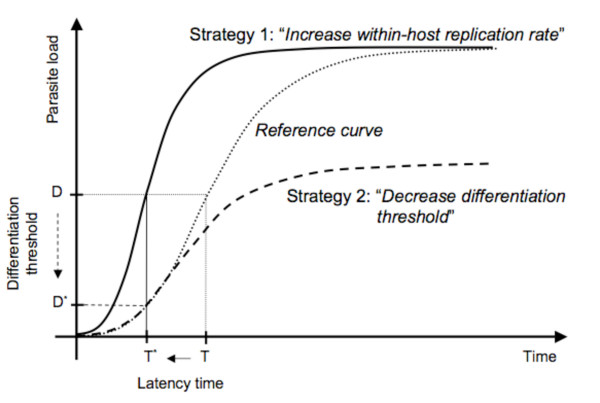
**Graphical representation of a simple theoretical model illustrating two different possibilities to evolve shorter latency**. Latency denotes the time until the onset of conversion of reproductive forms into transmission stages (infectious forms). Suppose a hypothetical ancestral parasite with latency time *T *(dotted line). Reproductive forms make 1 division per unit time. At a density-dependent differentiation threshold (*D *= 100 reproductive forms), 60% of the reproductive forms are converted into infectious forms at each bacterial generation (infectious forms do not multiply). Shorter latency time (T*) can evolve in two ways. Strategy 1: Faster replication of reproductive forms (solid line) reduces the time to the differentiation threshold *D*, thereby reducing latency time (T*). Consequently, maximum parasite load is also reached earlier. Strategy 2: Replication rate of reproductive forms remains unchanged (dashed line), but the differentiation threshold is reduced (D*). This shortens latency time (T*), but also reduces parasite load. If parasite load correlates with consumption of host resources and thus virulence (graphically, the area under the curve), strategy 1 is associated with higher virulence, whereas strategy 2 is associated with lower virulence. NB: Hosts do not divide in this model; host mortality is nil.

We used experimental populations of the bacterial parasite *Holospora undulata *and its protozoan host, *Paramecium caudatum*, to investigate effects of early vs. late killing on different aspects of parasite life history. The life cycle of infection [26] is similar to that of certain bacteria-phage systems in that it involves within-host replication with vertical transmission when its host divides and horizontal transmission. However, unlike, e.g., in lytic phages, horizontal transmission does not require host death. Furthermore, this parasite produces two morphologically and functionally distinct forms: reproductive forms for within-host growth and infectious forms for horizontal transmission. Reproductive forms cannot be transmitted horizontally and infectious forms cannot replicate. The conversion of reproductive into infectious forms seems to rely on a density-dependent switch [27], similar to other parasites, such as *Legionella *[28] or Trypanosomes [29]. These features give such parasites evolutionary options to respond to shortened host life span that do not necessarily lead to increased virulence. For example, earlier onset of horizontal transmission may be achieved by lowering the threshold density that triggers the production of infectious forms. This can be done without changing within-host growth rate; moreover, if conversion into infectious forms is irreversible, total parasite load will be lower and thus virulence will be lower (Figure 1).

We experimentally manipulated host life-span in a serial transfer experiment, by killing hosts at two time points. In the *early-killing *treatment, hosts were killed 11 days after infection and infectious forms of the parasite harvested to start a new infection cycle on previously unexposed hosts. Initially, only a small fraction of the infected hosts produced infectious forms at this time point. In the *late-killing *treatment, hosts were killed after 14 days, when the majority of infected hosts had begun to produce infectious forms. After 13 infection cycles, we compared parasites from the two treatments for their infectivity, latency time (= time to production of infectious forms), parasite load and virulence. We further examined correlations between these traits among parasite selection lines.

## Results

Adaptation assays were performed on evolved parasite lines from the *early*- and *late-killing *treatments. Ancestral parasite lines had been stored at -80°C, but their extremely low infection success after thawing precluded the reconstruction of the parasite founder population for the assay.

### Infectivity

In a dose-controlled inoculation experiment, mean infectivity of parasite selection lines ranged from 18–69%, except for one outlier from the *late-killing *treatment (2%). On average, parasites from the *late-killing *treatment tended to be more infectious (percentage of infected hosts, without the outlier: 42.1 ± 5.3% S.E.) than those from the *early-killing *treatment (30.7 ± 3.7%), although the difference was not statistically significant (F_1,18 _= 3.05, p = 0.0978; with the outlier: p > 0.4).

### Latency time

**Figure 2:**
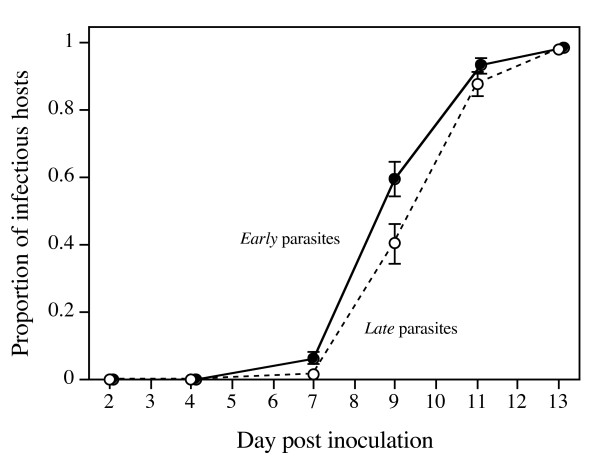
**Proportion of infected hosts producing infectious forms over the course of 13 days after inoculation**. A higher proportion of infectious hosts indicates a shorter latency time. Values for parasites from *early*- and *late-killing *treatments were averaged over selection line means. Error bars represent standard error.

To follow the subsequent development of infection, we sampled infected assay populations at different time points over the course of two weeks. The first hosts producing infectious forms were observed on day 7 after inoculation (1.6% of all infected individuals; all other infections were still at the reproductive stage). The proportion of such infectious hosts was significantly higher in populations infected with parasites from the *early-killing *treatment than in populations infected with parasites from the *late-killing *treatment (MANOVA on the proportion of infectious forms bearing individuals, day 7–13: F_1,19 _= 6.38, p = 0.0212). This effect was most pronounced on day 7 (ANOVA: F_1,18 _= 5.41, p = 0.0320) and on day 9 (F_1,18 _= 5.48, p = 0.0249), when there was a 20% difference in the proportion of infectious hosts (Figure 2). From logistic curve fits, we estimated the time until 50% of the hosts in a population were producing infectious forms. On average, parasites from the *early-killing *treatment had reached this point more than half a day earlier (8.81 ± 0.15 d) than parasites from the *late-killing *treatment (9.35 ± 0.19 d; treatment effect: F_1,18 _= 5.35, p = 0.0328). Thus, *early *parasites had a shorter latency time.

### Parasite within-host growth and production of transmission stages

#### First week after infection

**Figure 3:**
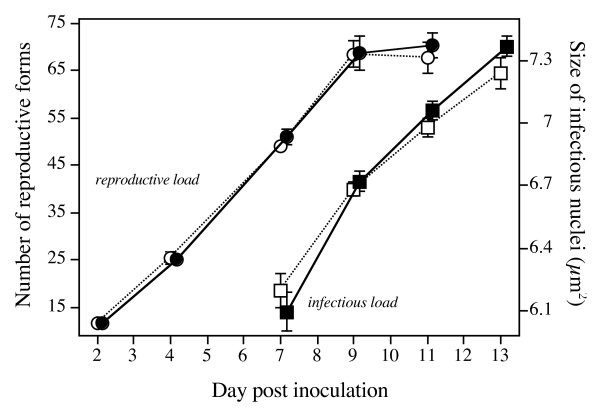
**Reproductive and infectious parasite loads over the course of 13 days after inoculation**. Reproductive loads (left axis) represent the number of reproductive forms per infected individual; infectious loads (right axis) were estimated from the size (length × width) of nuclei carrying infectious forms. Means for parasites from *early*- and *late-killing *treatments were calculated by averaging over individuals within assay tubes, then over assay tubes and selection lines. Error bars represent standard error.

Parasite loads increased from 11.5 (± 0.4) reproductive forms on day 2 to 51.1 (± 1.2) on day 7. Given one division of the host, reproductive forms made 3–4 doublings during this period. One possibility for parasites from the *early-killing *treatment to shorten their latency time is through faster within-host replication (Figure 1). Indeed, the mean reproductive parasite load on day 7 was positively correlated with the proportion of individuals producing infectious forms on day 9 (across all assay tubes: r = 0.40, n = 62, p = 0.0013); thus, the accumulation of reproductive forms in the micronucleus was a good predictor of the onset of the production of infectious forms. This is consistent with the idea of a density-dependent switch from the reproductive to the infectious stage, and thus indicates that faster within-host replication reduces latency time (see also Table 1). On day 7, reproductive loads of parasites from the *early-killing *treatment were, on average, 5% larger than those of parasites from the *late-killing *treatment, but this difference was not significant (MANOVA day 2–7: killing treatment: F_1,19 _= 0.10, p = 0.7563; treatment × time interaction: F_2,18 _= 0.26, p = 0.7764; Figure 3). Thus, there was no statistical support for faster within-host replication of parasites from the *early-killing *treatment.

**Table 1:** Pearson correlation coefficients for five parasite traits.

Trait		Reproductive load	Latency time	Infectious load	Virulence
Infectivity	*Early*	+0.38	-0.56(*)	+0.21^(a)^	-0.40
	*Late*	-0.12	+0.12	+0.45^(a)^	+0.34
	*Overall*	+0.02	+0.08	+0.29	-0.04
Reproductive load (day 7)	*Early*		**-0.30**^a^	+0.44^(a)^	-0.75*^a^
	*Late*		-0.80**^a^	+0.45^(a)^	**-0.34**^a^
	*Overall*		-0.55*	+0.47*	-0.38(*)
Latency time	*Early*			**-0.61**(*)^a^	-0.07^(a)^
	*Late*			**-0.55**(*)^a^	+0.51^(a)^
	*Overall*			-0.58**	+0.01
Infectious load (day 13)	*Early*				-0.15
	*Late*				+0.09
	*Overall*				+0.14

Correlations were based on the means per parasite selection line and calculated across selection lines within treatments (*early-killing*, *late-killing*) and for all selection lines pooled (*overall*). Within-treatment tests were complemented by Analyses of Covariance (ANCOVA), with treatment as a cofactor, one parasite trait as covariate (line) and another (column) as response variable. For definition of traits, see *Methods*. Significant correlations marked in bold (uncorrected for multiple testing). Note: ANCOVA can reveal significant relationships, while correlation tests within treatments are non-significant.

(*) p < 0.10; * p < 0.05; ** p < 0.01; main effect of covariate in ANCOVA: ^(a) ^p < 0.09; ^a ^p < 0.05

#### Second week after infection

Shorter latency can also be achieved by decreasing the within-host density threshold. In this case, our model predicts smaller reproductive loads once differentiation into infectious forms has set in (Figure 1). However, when comparing the fraction of hosts still in the exclusively reproductive state, we found no significant difference in reproductive loads between parasites from *early*- and *late-killing *treatments (MANOVA days 7–11: F_1,17 _= 0.20, p = 0.6604). If anything, *early *parasites produced more, rather than fewer, reproductive forms than did *late *parasites (Figure 3).

During the second week after infection, infectious forms accumulated in the infected micronculei (Figure 3). Accumulation occurred more rapidly in the hosts infected with parasites from the *early-killing *treatment, resulting in an approx. 10% difference in parasite load between the two treatments on day 13 (F_1,19 _= 3.20, p = 0.0896; Figure 3). Overall, earlier onset of the production of infectious forms was associated with larger quantities of infectious forms at the end of the second week (r = 0.41, n = 61, p = 0.0010).

### Host density and virulence

#### Assay populations (days 2–13 after infection)

Overall, host population density in infected assay tubes was lower than that in the uninfected controls (MANOVA: F_1,64 _= 5.61, p = 0.0209), with an average reduction of 20–25% during the last 3 assay dates (day 9, 11, 13). Population density did not significantly differ between tubes infected with parasites from *early*- and *late-killing *treatments (F_1,19 _= 0.61, p = 0.4446).

#### Individual experiment (20–40 d after infection)

**Figure 4:**
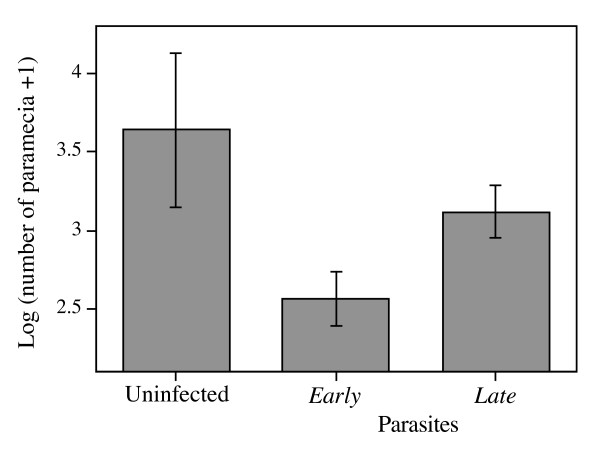
**Final host density in the individual assay**. Densities measured 20 days after isolation of single individuals. Means for parasites from *early*- and *late-killing *treatments were based on selection line means; means for uninfected controls were taken across all assay tubes. Error bars represent standard error.

In an additional experiment, we measured clonal growth and survival of infected individuals isolated from the assay populations. After 20 days of culture, we obtained a significant treatment effect on final host density (F_2,19 _= 4.29, p = 0.0290; Figure 4). Like in the assay populations, densities were higher in uninfected than infected lines. Moreover, lines infected with parasites from the *early-killing *treatment had significantly lower densities than those infected with parasites from the *early-killing *treatment (contrast '*early *vs. *late*': F_1,19 _= 5.65, p = 0.0285; Figure 4). This effect was mainly due to a strong difference in survival: the mortality of entire lines was 32.0 ± 3.8% for *early *parasites, but only 17.8 ± 4.4% for *late *parasites (F_1,19 _= 9.21, p = 0.0068).

### Correlations

**Figure 5:**
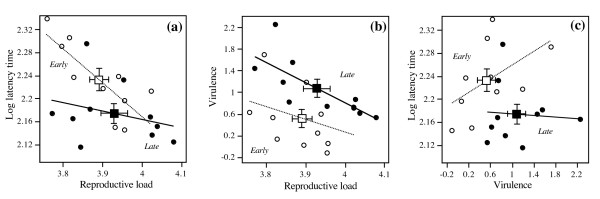
**Within- and between-treatment relationships between mean reproductive parasite loads, latency time and virulence**. Each circle represents the mean for a single parasite selection line, averaged over three replicate assay tubes. Black circles denote selection lines from *early-killing*, open circles selection lines from *late-killing *treatments. Regression lines illustrate correlations within treatments. Squares indicate the treatment means, averaged over selection line means. Error bars represent standard error. Parasite load = number of reproductive forms per infected individual on day 7; latency time = time until 50% of infected hosts produce infectious forms; virulence = Log-transformed host density uninfected minus log-transformed host density infected in the individual assay (i.e., larger positive values indicate higher virulence).

Correlations between five characters were analyzed (Table 1). When pooling the means of selection lines from the two treatments, we obtained statistically significant overall correlations between latency time and reproductive or infectious parasite loads, with relatively large effect sizes (note that we did not correct p-values for multiple testing [30]). Selection lines with a higher reproductive load (indicating higher within-host replication during the first week) had a shorter latency time (Figure 5a) and larger infectious parasite loads. Shorter latency was also associated with higher final loads. In contrast with the positive association between treatments means, reproductive load was negatively correlated with virulence: selection lines with a faster accumulation of reproductive loads during the first week after infection tended to be less virulent later on (Figure 5b). These correlations also held across selection lines within treatments (Table 1). The remaining overall correlations had small effect sizes (|r| < 0.3), and in several cases, the within-treatment correlations differed in sign and magnitude (illustrated for latency time and virulence in Figure 5c).

## Discussion

Life-history theory predicts that cutting off future reproduction selects for increased investment in early reproduction [18]. Consistent with this prediction, parasites from the *early-killing *treatment (where hosts were killed 11 days after infection) had a shorter latency and accumulated more transmission stages in the micronuclei of infected hosts than did parasites from the *late-killing *treatment (where hosts were killed 14 days after infection). *Early *parasites were also more virulent, in agreement with theoretical models predicting that higher virulence evolves under increased background mortality [11,15].

### What causes shorter latency time?

We had hypothesized that parasites, such as *H. undulata*, may reduce latency time by lowering the threshold within-host density that triggers the conversion of reproductive into infectious forms. However, we found no evidence for a lower conversion threshold. In contrast with the predictions under this hypothesis (Figure 1), parasites from the *early-killing *treatment did not have lower parasite loads, nor were they less virulent than *late *parasites.

Alternatively, parasites could reduce their latency time by increasing the rate at which they replicate. If the conversion threshold does not change, the more rapid replication would imply earlier conversion (Figure 1). The negative relationship between the densities of reproductive forms after the first week and latency time indicates such a link in our experiment, but there was no clear-cut evidence for faster within-host replication of early parasites. First, the mean density of reproductive forms of early parasites was only marginally larger than that of late parasites after the first week, and this difference was not statistically significant. Second, at later stages of infection, our measure of parasite load (i.e., the size of the infected nucleus) does not distinguish between reproductive and infectious forms. Therefore, the higher loads of early parasites during the second week may simply reflect their shorter latency time and the concomitant earlier accumulation of infectious forms, irrespective of the rates of replication of reproductive forms. A more conclusive analysis of the within-nucleus dynamics will require a finer temporal resolution of measurements of reproductive loads during the first week after infection and improved techniques allowing us to quantify reproductive forms at later stages of infection.

### Virulence: population versus individual assays

Parasites from the *early-killing *treatment were more virulent in the assay of individual *Paramecium*, whereas there was no significant treatment effect in the population assay. Several explanations are possible for this discrepancy. First, host density is strongly reduced only when infectious forms massively accumulate in the infected host [31]. At the time of our final population measurements, although infection was beginning to reduce host population density, parasite loads were moderate. Thus, it was perhaps too early to see a treatment effect, in particular as only a fraction of the populations was infected. Indeed, the test of individuals was performed later on, when infections could even be recognized under the dissecting microscope. Second, in our selection protocol, we killed the entire host population to prepare the inoculum for a new infection cycle. Although this protocol selected for more rapid production of infectious forms, it may have relaxed the pressure for the parasite to kill its host to be transmitted horizontally. As selection for virulence is only relaxed at the time of harvest, an effect of treatment could still be observed at later stages of infection, in the assay of individuals, and thus represent a delayed cost of virulence [32].

### Correlations between parasite life-history traits

Life-history theory relies on evolutionary trade-offs between traits that influence fitness [18]. In host-parasite interactions, the most prominent trade-off is between transmissibility and virulence [3,33,34]. In our experiment, the relationships between the means of the *early*- and *late-killing *treatments suggested trade-offs between two components of transmissibility (latency time and infectivity) and virulence. We may interpret our results as correlated sets of evolutionary responses to the different selection pressures imposed by the experimental treatments, leading to separate peaks in an adaptive landscape, possibly determined by a limited number of genes of large effect, as frequently observed in studies on experimental evolution [35].

In part, however, this picture contrasts with the patterns of quasi-genetic correlations observed between the means of individual selection lines (rather than between the two treatment means). Thus, if the means of the selection lines rather than the means of the two treatments were analyzed, there was no evidence a for negative correlation between latency time and virulence among selection lines (Figure 5c), reproductive load (and thus presumably within-host replication rate) was negatively correlated with virulence among selection lines within treatments (Figure 5b) (despite a positive correlation between treatment means), and infectious load showed no clear relationship with virulence. These patterns are the opposite of the assumptions of basic theories [34] and differ from results of other studies investigating the experimental evolution of these relationships [24,36,37].

To some extent, the scatter around the treatment means may be non-genetic; we offer three pieces of support for this view. First, we measured parasite loads and virulence in different individuals and at different time points, possibly introducing experimental noise that blurred the underlying genetic correlations. Second, our parasite is not an obligate killer, so that there may be a less straightforward (genetic) link between latency time and virulence than there is between lysis time and virulence in obligately killing bacteriophages [14]. Third, as mentioned above, our experimental protocol may have uncoupled selection pressures on virulence and on latency, allowing the traits to evolve independently and genetically unlinked, at least within the boundaries of the local area around the adaptive peaks. However, the scatter may also represent residual genetic variation, possibly due to genes of minor effect. This variation may be transient. Our results would thus show a snapshot of these lines on their evolutionary pathway towards the adaptive peaks. Alternatively, our snapshot shows individual selection lines moving away from, rather than towards, the adaptive peak. This may reflect additional processes of selection, generating new relationships, such as the negative within-treatment correlations between reproductive load and virulence (Figure 5b). In this case, parallel to the large-scale divergence of treatment means of parasite load and virulence towards different adaptive peaks, local-scale selection may be operating to compensate costs of virulence.

Without more detailed genetic analysis of the evolved lines and the localization of the ancestral parasite population in the adaptive landscape, these interpretations remain speculative. Nonetheless, our results illustrate that the sign of relationships within and among groups or treatments can be different and that interpretations based solely on treatment means can be incomplete or even misleading.

### Realism

The point of our study was to test an evolutionary idea, namely the role of background host mortality in parasite evolution, using a relatively controlled experimental setup. We acknowledge that scenarios of selection in natural populations may be more complex than those in this or related experiments. For example, depending on the source(s) of mortality, selection may not exclusively act on the parasites that are culled from the population [22], nor exclusively on those that remain in the population after culling [24]. Moreover, the selective advantage of shorter-latency genotypes critically depends on epidemiological factors, namely the density of available hosts [38]. So far, however, experimental studies on extrinsic host mortality have not allowed entirely free action of epidemiological dynamics: In our case, we did not interfere with these dynamics prior to the mortality event, but after the event, parasites were provided with new, naïve hosts *ad libitum *[see also [22,23]]; Ebert and Mangin [24] simply replaced infected hosts with uninfected hosts. Relaxing these experimental constraints may substantially alter evolutionary outcomes. If the parasites from culled hosts remain in the population (e.g., by adding parasites from dead hosts back into the population), the force of infection and levels of coinfection will change. Furthermore, if dead hosts are not artificially replaced, mortality may be compensated by increased birth rates. In our system, for example, increased mortality would be coupled with increased levels of vertical transmission during population re-growth. Thus, selection on the efficacy of vertical and horizontal transmission, as well as evolutionary responses in the host, may act simultaneously (Magalon, Nidelet and Kaltz, unpubl. data). In other words, under more realistic scenarios, it may be difficult to respect the all-else-being-equal rule, and manipulating one factor in question (host mortality) may inevitably produce changes the epidemiology and population dynamics that are not necessarily anticipated by the standard models [24].

## Conclusion

Our study demonstrated an adaptive shift in parasite age at maturity (i.e., latency time), consistent with basic life-history theory. As predicted by standard models of virulence evolution, experimentally shortened host life span lead to an evolutionary increase in virulence, confirming results from studies on viral pathogens [22,23]. Faster within-host replication is the most likely explanation for the shorter latency time, although this needs to be confirmed by more detailed analysis. However, conclusions about the evolution of genetic correlations or trade-offs between parasite traits should be taken with caution. More generally, our results illustrate how variation in environmental conditions affecting host life-history can feed back on the evolution of parasite life-history. The remaining challenges are experiments testing more realistic scenarios, relaxing constraints on epidemiological and (co)evolutionary processes.

## Methods

### Study organisms

*Paramecium caudatum *is a freshwater ciliate [39], with predominant asexual reproduction (mitotic division). The diploid micronucleus is similar to a germ line and active mainly during the sexual cycle.

The gram-negative *Holospora undulata *(alpha-group of the Protobacteria [40]) infects the micronucleus of its host. Infection starts with the uptake of infectious forms (10–15 μm) from the water. The infectious forms escape from the digestive vacuole and mediate their transfer to the micronucleus. Within 24 h, a single infectious form differentiates into 4 reproductive forms (5 μm) that will then start multiplying (initially ca. 1–2 doublings per day). During the first week after infection, only reproductive forms are produced [41]. When bacterial loads further increase, reproductive forms differentiate into infectious forms; possibly, differentiation is triggered by a density-dependent threshold. Infectious forms are released into the environment during mitotic cell division of the host (reproductive forms are transmitted vertically to daughter cells) or upon host death. Accumulating parasite loads produce a heavily swollen micronucleus, filling out the entire individual and packed with several hundreds of infectious forms. At this point, infection strongly reduces cell division and survival of the host [31].

### Experimental protocol

A clonal mass culture [K8, [42]] was infected with a mix of parasites from six selection lines from another long-term experiment [43]. From this mass culture, we created replicate selection lines, each consisting of ca. 10^4 ^individuals in a 50 ml plastic tube, filled with 35 ml of culture medium (1 g dried organically grown lettuce, ground with mortar and pestle, then autoclaved in 1.5 l of Volvic™ mineral water [Groupe Danone, France], and inoculated with the bacterium *Serratia marcescens *[strain A173, Institut Pasteur, Paris, France], as food resource for the paramecia).

Replicate lines were randomly assigned to two treatments. In the *early-killing *treatment, individuals were killed and infectious forms harvested 11 days after inoculation; in the *late-killing *treatment, infectious forms were collected 14 days after inoculation. The motivation for our experimental treatments is as follows. First, in the *early-killing *treatment we intended to impose strong selection on latency time (= onset of production of infectious forms), by killing the population and harvesting the parasite at a time when only few hosts produced infectious forms. In previous experiments [42] as well as in a preliminary test (T. Nidelet, unpubl. data), the frequency of infectious hosts (i.e., carrying at least 1 infectious form) on day 11 ranged from 10 to 30%. Second, in the *late-killing *treatment, we intended to relax selection on latency time, by killing the population when most infected hosts were already producing large numbers of infectious forms. In most experiments, up to 90% of the infected hosts were highly infectious on day 14 [e.g., [42]]. We did not choose an even later killing time to avoid potentially confounding selection for parasites with an extremely short latency, which might have allowed them to complete a second infectious cycle at the time of the *late-killing *harvest.

To harvest infectious forms, 30 ml of a tube (5 ml were kept as a backup) were centrifuged for 20 min at 1500 g, 25 ml of the supernatant removed and 1 ml of dimethyl sulfoxide (Sigma, France) was added to kill the paramecia. After two rounds of washing (centrifugation for 20 min at 1500 g, replacement of supernatant with sterile Volvic), a final volume of 1.5 ml was vortexed for 30 sec in a 2 ml plastic tube, together with several 2-mm glass beads, to grind up the dead paramecia. Thus, the inoculum contained a mix of naturally released and extracted infectious forms.

Approximately 10^5 ^infectious forms were used to start a new infection cycle, with hosts from the unselected base culture. Paramecia were concentrated in a volume of 5 ml (by centrifugation for 20 min at 300 g) before adding the inoculum. With this protocol, most infections occur during the first 24 h; multiply infected hosts are rare (T. Nidelet, unpubl. data). After 48 h, we added 30 ml of fresh medium, allowing the populations about one doubling until the end of the infection cycle.

Thirteen infection cycles were performed for each of 11 replicate selection lines in each treatment. On three occasions in the *early-killing *treatment group (cycles 3, 7, 11), the prevalences in certain experimental populations were relatively low. By killing these populations on day 11, we might have harvested an insufficient number of infectious forms to start a new infection cycle. To avoid this problem, we only killed (all) *early-killing *selection lines on day 14, when infected individuals were producing large amounts of infectious forms. Thus, the *early-killing *treatment consisted of 13 *early-killing *rounds and three intermittent *late-killing *rounds.

### Adaptation assay

During a 14th infection cycle, we compared parasites from *early*- and *late-killing *treatments, with three replicate assay tubes per selection line. Three additional assay tubes received a mock inoculum and served as controls. Reconstruction of the parasite founder population from the frozen ancestral lines was not possible because of extremely low infection success of the inocula after thawing.

On day 2, 4, 7, 9, 11 and 13 after inoculation, we measured the density of paramecia by sampling 4 × 100 μl from each tube; ca. 60 individuals were fixed with lacto-aceto-orcein [41] to determine the proportion of infected individuals (at 1000× magnification, phase contrast). For each infected individual we recorded the absence or presence of infectious forms in the micronucleus. On days 2 and 4, we counted the number of reproductive forms in the micronuclei. Later on, we took the dimensions of the infected micronucleus (length × width in μm) as an estimate of parasite load. During the first week after infection, this measure gives a good approximation of the number of reproductive forms in the micronucleus; at later stages of infection, it mainly reflects the number of infectious forms [27].

On day 20 after infection, we started an additional experiment to measure parasite virulence. We isolated five infected individuals from each infected assay tube (except for five assay tubes that were omitted due to handling error) as well as five uninfected individuals from each control assay tube. Individuals were placed singly in 200 μl of fresh medium in 500 μl plastic tubes, with fresh medium (100 μl) added once, on day 10 after isolation. On day 20 after isolation, we determined the density of paramecia in each tube. The density in infected assay tubes relative to uninfected controls is a measure of parasite virulence, integrating effects of infection on host division and survival.

### Statistical analysis

Infections with one parasite selection line from the *early-killing *treatment failed to develop for unknown reasons. We had observed normal development of this line in all previous infection cycles and therefore decided to exclude the three assay tubes of this selection line from the analyses. Of the remaining 63 inoculated assay tubes, one was found uninfected and grouped with the three control assay tubes. For these tubes, we analyzed treatment effects on the following traits:

i) Infectivity: the proportion of infected hosts, combined over days 2, 4 and 7.

ii) Parasite load: in the reproductive state, parasite load was calculated as the mean number of reproductive forms for hosts not producing infectious forms; parasite load in the infectious state was estimated as the mean nucleus size [μm^2^] of hosts producing infectious forms.

iii) Latency time: For each assay date, we calculated the proportion of infectious individuals (i.e., that produced infectious forms) in a population; this proportion reflects the timing of the onset of the production of infectious forms and thus latency time. We also used logistic curve fits to estimate the time until 50% of the infected individuals produced infectious forms. This time was taken as a correlate of latency time.

iv) For effects of infection on host growth and survival (virulence), we compared final host population size in the assay tubes (day 13 after infection) and final densities in the assay of individual paramecia (day 20 after isolation). Virulence in the individual assay was calculated as: Log(mean density +1, uninfected) – Log(mean density +1, infected); this difference was calculated for each replicate, then averaged over assay tubes and parasite selection lines. Note that for 4 assay tubes (from 4 different parasite selection lines) in the individual assay, we had established fewer than the required five replicates; to obtain reliable estimates at the level of the assay tube, these replicates (n = 10) were excluded from analysis.

Using the JMP statistical package [44], we carried out nested ANOVAs for single assay dates, with killing treatment and selection line(within killing treatment) as explanatory variables. We also report results from repeated-measures MANOVAs, carried out on the averages over the three replicate tubes per parasite selection line. Where necessary, we used arcsine transformation of proportions and log-transformations of parasite load to meet the assumptions of normality and homogeneity of variance. Finally, we examined pairwise correlations between the above traits. Correlations were analyzed across all selection lines pooled, as well as across selection lines within treatments (the latter complemented by Analyses of Covariance).

## Authors' contributions

TN carried out the selection experiment and the adaptation assay and participated in the experimental design of the study, statistical analysis and drafting of the manuscript. JC participated in the experimental design of the study and the drafting of the manuscript. OK participated in the experimental design of the study, the preparation of the selection experiment, statistical analysis and the drafting of the manuscript. All authors read and approved the final manuscript.
